# Telomeric repeats act as nucleosome-disfavouring sequences *in vivo*

**DOI:** 10.1093/nar/gkt1006

**Published:** 2013-10-29

**Authors:** Yuichi Ichikawa, Nobuyuki Morohashi, Yoshifumi Nishimura, Hitoshi Kurumizaka, Mitsuhiro Shimizu

**Affiliations:** ^1^Laboratory of Structural Biology, Graduate School of Advanced Science and Engineering/RISE, Waseda University, 2-2 Wakamatsu-cho, Shinjuku-ku, Tokyo 162-8640, Japan, ^2^Program in Chemistry and Life Science, School of Science and Engineering, Department of Chemistry, Graduate School of Science and Engineering, Meisei University, 2-1-1 Hodokubo, Hino, Tokyo 191-8506, Japan and ^3^Graduate School of Medical Life Science, Yokohama City University, 1-7-29 Suehiro-cho, Tsurumi-ku, Yokohama 230-0045, Japan

## Abstract

Telomeric DNAs consist of tandem repeats of G-clusters such as TTAGGG and TG_1-3_, which are the human and yeast repeat sequences, respectively. In the yeast *Saccharomyces cerevisiae*, the telomeric repeats are non-nucleosomal, whereas in humans, they are organized in tightly packaged nucleosomes. However, previous *in vitro* studies revealed that the binding affinities of human and yeast telomeric repeat sequences to histone octamers *in vitro* were similar, which is apparently inconsistent with the differences in the human and yeast telomeric chromatin structures. To further investigate the relationship between telomeric sequences and chromatin structure, we examined the effect of telomeric repeats on the formation of positioned nucleosomes *in vivo* by indirect end-label mapping, primer extension mapping and nucleosome repeat analyses, using a defined minichromosome in yeast cells. We found that the human and yeast telomeric repeat sequences both disfavour nucleosome assembly and alter nucleosome positioning in the yeast minichromosome. We further demonstrated that the G-clusters in the telomeric repeats are required for the nucleosome-disfavouring properties. Thus, our results suggest that this inherent structural feature of the telomeric repeat sequences is involved in the functional dynamics of the telomeric chromatin structure.

## INTRODUCTION

In eukaryotic chromosomes, the nucleosome is a fundamental structural and functional unit, composed of a histone octamer (two molecules each of histones H2A, H2B, H3 and H4) and 146 bp of DNA (1,2). The ends of linear chromosomes are called telomeres, and they play many important structural and functional roles in genome stabilization and maintenance (3–5). Telomeric DNAs consist of tandem repeats of 5–8 bp. In human and yeast, the repeat sequences are TTAGGG and TG_1__–3_, respectively, and these G-rich strands are extended to form a 3' single strand tail (6). There are characteristic differences in telomere organization between lower and higher eukaryotes (3–5). The lengths of the telomeric repetitive elements are relatively short, and are typically a few hundred base pairs in yeasts and other lower eukaryotes. In contrast, the repetitive elements in higher eukaryotes are several thousand base pairs long, and their lengths are highly variable. Furthermore, in lower eukaryotes, the telomeric tandem repeats are organized within a non-nucleosomal chromatin structure, in which they are covered by an array of Rap1 and other protein components (3,4,7). In contrast, the telomeric repeats in higher eukaryotes are organized within tightly packaged nucleosomes (8–15), and the TRF1 and TRF2 proteins, which directly bind to telomeric repeat sequences (16–18), form a complex with nucleosomes (19–24). Thus, the organization of the chromatin structures in the telomeric repeat regions is remarkably different between lower and higher eukaryotes.

Although histones are non-specific DNA binding proteins, nucleosome formation is influenced by DNA sequences and their structural properties. For example, some repeating sequences, such as (A)_n_ · (T)_n_, (CG)_n_ · (GC)_n_ and (CGG)_n_ · (GCC)_n_, block nucleosome formation, and their insertions strongly perturbed the positioning of nucleosome arrays in the yeast genome as well as in minichromosomes (25–27). In contrast, the human α-satellite DNA and the 601 sequence form stable nucleosomes, which have been analysed in detail by X-ray crystallography (28,29). Regarding telomeric repeat sequences, the free energies of nucleosome formation have been extensively analysed by competitive reconstitution assays of nucleosomes *in vitro*, which revealed that the human and yeast telomeric repeats have similar binding properties. Their affinities for the histone octamers were the lowest among the various sequences tested (4,30–32). These results suggested that the telomeric repeats require more energy for nucleosome formation *in vitro*, as compared with nucleosomes containing non-telomeric sequences. Consistent with this feature, using a restriction enzyme assay and atomic force microscope (AFM) imaging, it was demonstrated that the human telomeric nucleosomes have a peculiar feature, in which they are highly intrinsically mobile under physiological conditions (33,34). These *in vitro* studies seemed to argue against the difference in the nucleosome organization in the telomeric repeats between humans and yeasts *in vivo*. Thus, telomeric nucleosome formation and its properties *in vivo* remain to be elucidated.

To examine the effect of telomeric repeats on nucleosome formation *in vivo*, we utilized a defined yeast minichromosome system, which consists of an array of positioned nucleosomes. We have shown that the formation of positioned nucleosomes in the minichromosomes is inhibited by the insertions of telomeric repeats, in a length-dependent manner. Thus, our results implied that both the human and yeast telomeric repeats share the common feature of disfavouring nucleosome formation *in vivo*.

## MATERIALS AND METHODS

### Plasmids and strains

**Figure 1.**
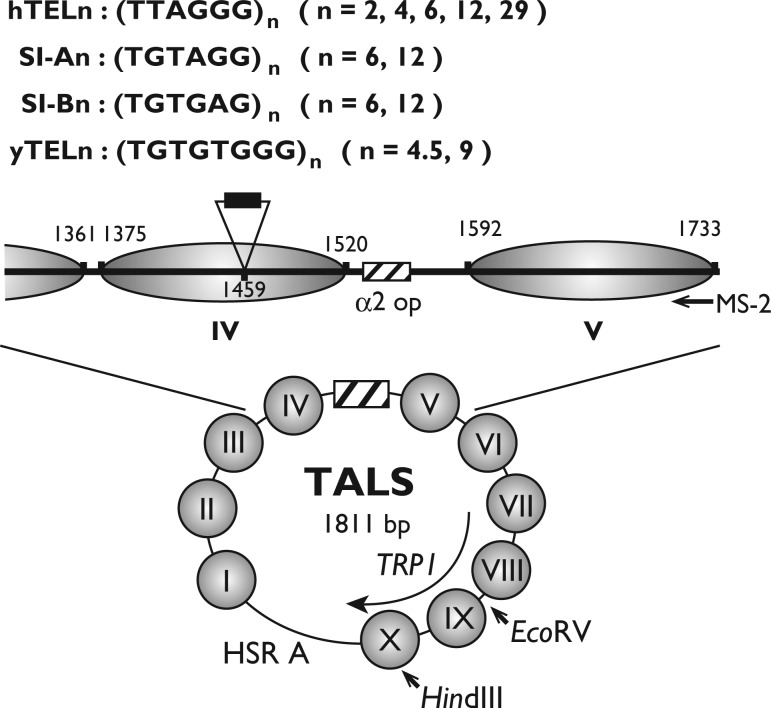
Chromatin structure of the TALS minichromosome in *MAT*α cells. Human or yeast telomeric repeat sequences (TTAGGG)_n_ (n = 2, 4, 6, 12 and 29, denoted as hTELn), (TGTGTGGG)_n_ (n = 4.5 and 9, denoted as yTELn) or the sequence isomers of the human repeat (TGTAGG)_n_ (n = 6 and 12, denoted as SI-An), (TGTGAG)_n_ (n = 6 and 12, denoted as SI-Bn), as indicated by the black box, were introduced into the *Sac*I site (1459 nt) in the centre of nucleosome IV (1375–1520 nt). The α2 operator is shown by the hatched box, and the positioned nucleosomes IV and V (1592–1733 nt) are shown by the grey ellipses. HSR A is the nuclease hyper-sensitive region A, containing the ARS1 region. The unique *Eco*RV (385 nt) and *Hin*dIII (615 nt) sites are indicated. The location of the MS-2 primer is indicated by the arrow.

A set of oligonucleotides bearing telomeric repeats [(TTAGGG)_n_, n = 2, 4, 6 and (TGTGTGGG)_n_, n = 4.5, 9] and the sequence isomers of the human telomeric repeat, [(TGTAGG)_n_ and (TGTGAG)_n_], with 3'-overhangs of *Sac*I sites were synthesized chemically, annealed to form the double-stranded fragments and ligated into the *Sac*I site of the vector TALS-pBR322ΔRI (26,35), forming the hTEL2-, hTEL4-, hTEL6-, yTEL4.5-, yTEL9-, SI-A6-, SI-A12-, SI-B6- and SI-B12-TALS-pBR322ΔRI plasmids, respectively. A set of oligonucleotides with (TTAGGG)_12_ and *Sac*I sites at the ends were synthesized chemically, annealed and ligated into the pTA2 vector, forming the pTA2-hTEL12 plasmid. The (TTAGGG)_12_ fragment was excised with *Sac*I and inserted into the TALS-pBR322ΔRI plasmid, to form hTEL12-TALS-pBR322ΔRI. The (TTAGGG)_29_ fragment was prepared as described (36), and the fragment was ligated with a *Sac*I adapter and inserted into the *Sac*I site of TALS-pBR322ΔRI, to form hTEL29-TALS-pBR322ΔRI. The locations of the inserts are schematically shown in Figure 1. All of the constructed plasmids were verified by DNA sequencing. The pBR322ΔRI portion was eliminated by a digestion with *Hin*dIII, and the TALS portions were self-ligated and transformed into the *Saccharomyces cerevisiae* strain AMP108 [*MATα ura3 trp1 leu2 lys2 ho::LYS2*] (37).

### Analysis of chromatin structure

The isolation of nuclei from yeast cells, the digestion of the nuclei with micrococcal nuclease (MNase) and the analysis of MNase cleavage sites by indirect end-label mapping were described previously (26,35,38,39). The MNase cleavage sites were also analysed by primer extension mapping at base pair resolution using the MS-2 primer (1735–1701 nt in TALS), as described previously (26,40) with a minor modification: the Ampdirect® for G/C-rich region (Shimadzu), instead of the 5× Taq polymerase buffer, was used for the primer extension. The nucleosome repeat assay was performed as described (26,38). An oligonucleotide with the sequence 5'-GTT TCA GAT ATG AAA ACT GTT GCA TTA TTG CCG TTC ATC ATT TTC GA-3' (bottom strand of 1513–1467 nt) was radioactively labelled by T4 polynucleotide kinase with [γ−^32^P] ATP, and used as the proximal probe (26). The DNA fragment of the nucleosome IX region (381–497 nt), which was prepared by polymerase chain reaction, was radioactively labelled using the BcaBest Labeling kit (TAKARA) with random primers, and used as the distal probe. The results from the indirect end-label mapping, primer extension mapping and nucleosome repeat assays were visualized with a Typhoon FLA 7000 biomolecular imager (GE Healthcare Life Sciences).

## RESULTS

### Experimental design

To examine the effects of telomeric repeat sequences on nucleosome formation *in vivo*, we designed various lengths of the repeats (Figure 1). (TTAGGG)_n_ (n = 2, 4, 6, 12 and 29) are human sequences, and are referred to as hTEL2, hTEL4, hTEL6, hTEL12 and hTEL29, respectively. (TGTGTGGG)_n_ (n =4.5 and 9) are yeast sequences, and are referred to as yTEL4.5 and yTEL9, respectively (their lengths are equivalent to those of hTEL6 and hTEL12, respectively). As control inserts, we also designed sequence isomers for the human telomeric repeat, in which the base composition in the repeat remained unchanged, but the sequences were altered. The two sequence isomers (TGTAGG)_n_ and (TGTGAG)_n_ (n =6 and 12) are referred to as SI-A6, SI-A12, SI-B6 and SI-B12, respectively. We inserted these repeats into the centre of the nucleosome IV region in the TALS vector (Figure 1) and introduced them into a *MAT*α strain for chromatin (the TALS minichromosome) assembly *in vivo*.

Prior studies showed that the TALS minichromosome consists of 10 positioned nucleosomes in *MAT*αcells (Figure 1) (35,40,41). The α2 operator is located in the region of 1535–1565 nt in TALS, and nucleosome IV is positioned both translationally and rotationally in the region of 1375–1520 nt adjacent to the α2 operator in TALS in *MATα* cells, but not in *MAT***a** cells. Nucleosome positioning by the α2 repressor depends on the Tup1 corepressor and the Isw2 chromatin remodelling complex (42–45), indicating that the nucleosomes are formed at the proper positions by the α2 repressor complex in an active manner. This minichromosome system has been utilized to examine the effects of specific DNA sequences such as poly dA·poly dT (26), as well as the binding of transcription factors, including Gal4, Bicoid and Hap1 (46–49), to their cognate sites within the nucleosome *in vivo*. In these studies, several DNA fragments, such as GC(GAGCTCGC)_6_ (50 bp), (CA)_17_ (34 bp) or the *CYC1* UAS1 (27 bp), which were inserted into the centre of the nucleosome IV region in the TALS vector, were incorporated in the positioned nucleosome IV (26,46). The nucleosomes are positioned independently of the sequences flanked by the α2 operator in both circular minichromosomes and **a**-cell-specific genes in the linear chromosomes (40,46,50,51), indicating that the chromatin assembly properties of the TALS minichromosome are comparable with those of the yeast genome. Thus, this system would be appropriate to examine the features of telomeric repeats for nucleosome assembly *in vivo*, even though telomeres constitute the ends of linear chromosomes.

### Effect of telomeric repeats on nucleosome positioning

**Figure 2.**
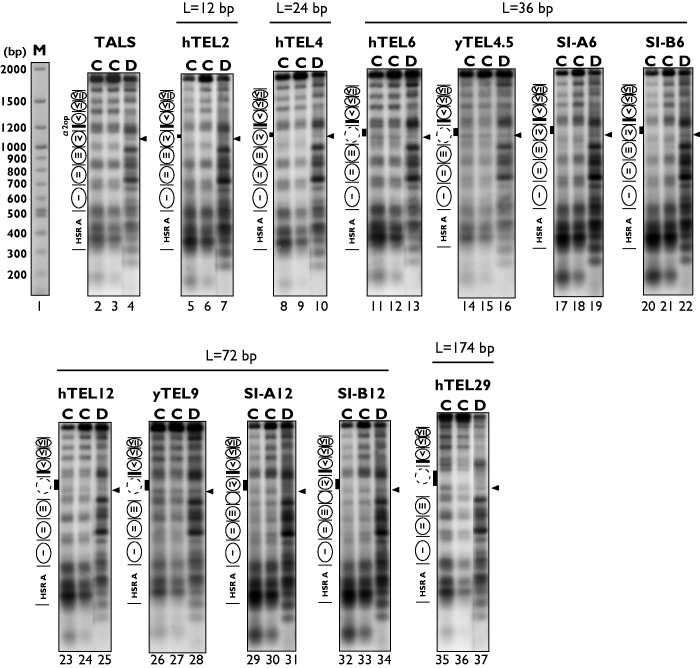
Indirect end-label mapping of MNase cleavage sites in TALS and its derivatives. The samples were digested with *Eco*RV and resolved by electrophoresis on a 1.2% agarose gel, and the samples were transferred to a nylon membrane for Southern blotting. The MNase cleavage sites were detected by indirect end-labelling using the *Eco*RV-*Hin*dIII fragment (385–615 nt in TALS; see Figure 1) as a probe (35,40,41). The locations of the nucleosomes and the α2 operator are illustrated on the left of the gel. Lanes labelled with ‘C’ indicate MNase digestion of isolated nuclei (chromatin) at two nuclease concentrations, and lanes labelled with ‘D’ indicate digestion of the naked DNA as a control. The lane labelled with ‘M’ indicates DNA markers, which were visualized by ethidium bromide staining on the same agarose gel used for the indirect end-labelling analysis. Solid and dotted circles indicate the positioned nucleosomes and the nucleosome disrupted by the inserts, respectively. The locations of the DNA inserts are indicated by black boxes. The characteristic MNase cleavage site near the inserts is indicated by arrowheads. For the TALS, hTEL2, hTEL4, hTEL6, hTEL12 and hTEL29 samples, each set of chromatin and naked samples was analysed on the same agarose gel, and the lanes were rearranged for clarity.

We analysed the chromatin structures of the TALS minichromosomes with or without telomeric repeats by limited digestion of nuclei with several different concentrations of MNase, which preferentially cuts between nucleosomes, and subsequent indirect end-label mapping (Figure 2). As indicated by a comparison of the MNase cleavage patterns between samples of chromatin as a minichromosome and its naked DNA (marked as ‘C’ and ‘D’ for chromatin and naked DNA, respectively), the TALS minichromosome without telomeric repeats clearly showed protected regions that occur periodically (Figure 2, lanes 2–4), which correspond to the locations of nucleosomes I∼VII. These results are consistent with previous studies (26,35,40,41). The cleavage patterns of the minichromosomes containing relatively shorter repeats, such as hTEL2 and hTEL4 (Figure 2, lanes 5, 6, 8 and 9), were similar to that of the TALS minichromosome (Figure 2, lanes 2 and 3). These results indicated that these shorter repeats were incorporated in the positioned nucleosome IV.

On the other hand, the MNase cleavage patterns at or near the nucleosome IV region of the hTEL6 minichromosome (Figure 2, lanes 11 and 12) were significantly different from those of the TALS minichromosome without telomeric repeats (lanes 2 and 3), and were closer to the cleavage pattern of the hTEL6 naked DNA (lane 13). The cleavage sites near the insert (indicated by the arrow in lanes 11–13 in Figure 2) were detectable in both the chromatin and naked DNA samples. In contrast, analyses of the sequence isomers with the same length (36 bp), as controls for hTEL6, revealed that this site was protected in the SI-A6 and SI-B6 minichromosomes (Figure 2, lanes 17, 18, 20 and 21). These results indicated that both sequence isomers were incorporated into nucleosome IV, but hTEL6 was not incorporated. The linker region between nucleosomes II and III in the hTEL6 minichromosome, as well as those in the SI-A6 and SI-B6 minichromosomes, appeared mostly unaffected, suggesting that the insertion of the hTEL6 repeat inhibits the formation of the positioned nucleosome IV, but not that of nucleosome III.

We compared the MNase cleavage patterns of the hTEL12 minichromosome with its sequence isomers, the SI-A12 and SI-B12 minichromosomes. For the SI-A12 minichromosome, the insert was protected from MNase digestion (Figure 2, lanes 29 and 30), as compared with that in the naked DNA (Figure 2, lane 31), indicating that the nucleosome IV was positioned. On the other hand, the MNase cleavage was inefficient within the hTEL12 and SI-B12 inserts (indicated by black boxes) in both the chromatin and naked DNA samples (Figure 2, lanes 23–25 and 32–34). Thus, the indirect end-labelling could not clarify whether nucleosome IV in the hTEL12 minichromosome is present between the α2 operator and the sites indicated by the arrow (Figure 2, lanes 23 and 24). It should be noted that, as judged by the MNase cleavage at the edges of nucleosome III, the region between nucleosomes III and IV became wider, and it appeared that one additional nucleosome was accommodated between nucleosomes III and IV in the SI-A12 and SI-B12 minichromosomes (Figure 2, lanes 29, 30, 32 and 33, marked with a white circle with no number). As a result, the position of nucleosome III in the minichromosomes containing SI-A12 or SI-B12 was shifted slightly towards nucleosome II, as compared with the original position of nucleosome III in the TALS minichromosome. Thus, although the assembly of nucleosome IV occurs independently of the sequences *per se* flanked by the α2 operator, the organization of an array of positioned nucleosomes in the minichromosomes can be affected by the lengths of the inserts.

Similar to the results obtained with the hTEL12 insert, the region of the hTEL29 insert was not cleaved by MNase in its chromatin and naked DNA samples (Figure 2, lanes 35–37), and thus it is not clear whether nucleosome IV is present in the hTEL29 minichromosome. Therefore, we conducted high-resolution mapping of the MNase cleavage sites by primer extension and nucleosome repeat assays, as described below. Together with the indirect end-labelling results, we will discuss the effects of these longer telomeric insertions on the positioning of nucleosome IV in the minichromosomes.

We also examined the effects of yeast *S. cerevisiae* telomeric repeats (yTEL4.5 and yTEL9), which were the same lengths as the hTEL6 and hTEL12 inserts, respectively. The alterations in the MNase cleavage patterns in the nucleosome IV region in the yTEL4.5 (Figure 2, lanes 14–16) and yTEL9 (Figure 2, lanes 26-28) minichromosomes were similar to those of the hTEL6 (Figure 2, lanes 11–13) and hTEL12 minichromosomes (Figure 2, lanes 23–25), respectively. Thus, both the human and yeast telomeric repeats have similar properties to alter nucleosome positioning in yeast cells *in vivo*.

### High-resolution mapping of MNase cleavage sites in the nucleosome IV region

**Figure 3.**
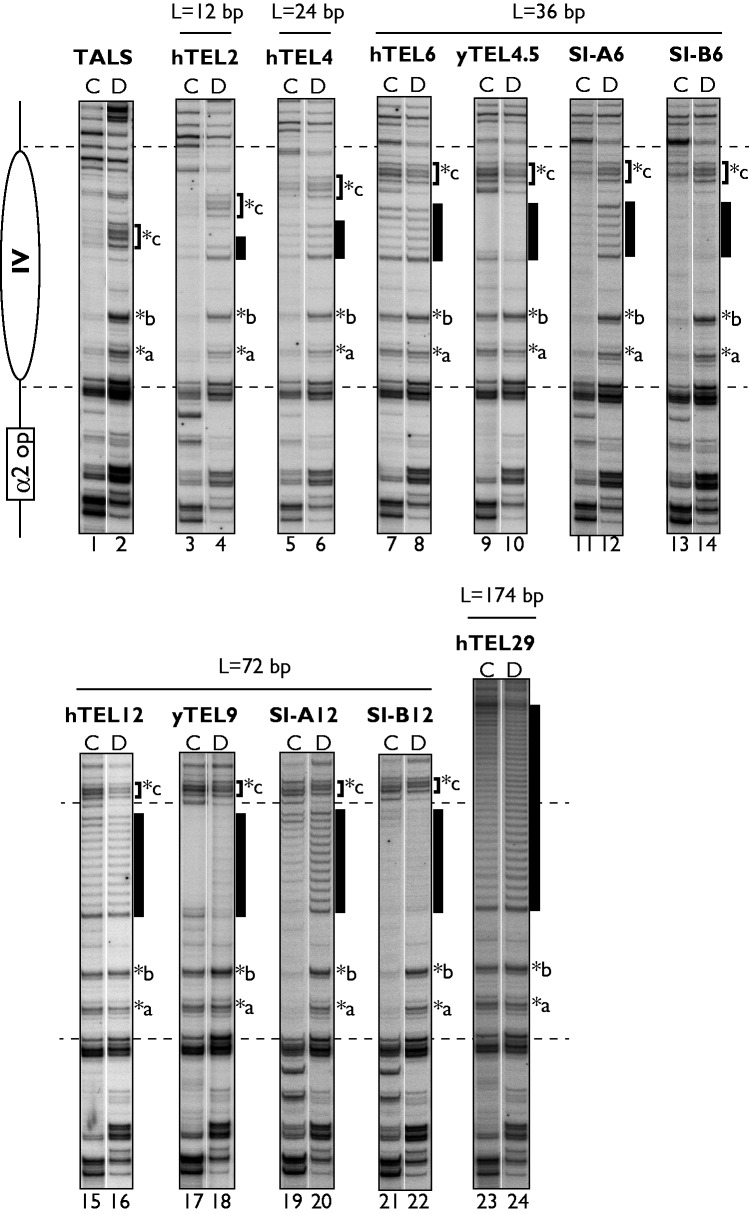
High-resolution primer extension mapping of MNase cleavage sites in the nucleosome IV regions in TALS and its derivatives containing human and yeast telomeric repeats or control inserts. Lanes labelled with ‘C’ indicate MNase digestion of isolated nuclei (C1 in Supplementary Figure S1), and lanes labelled with ‘D’ indicate digestion of the naked DNA (D in Supplementary Figure S1), as a control. The locations of the DNA inserts are indicated by black boxes. The characteristic MNase cleavage sites in the nucleosome IV region are indicated by asterisks. The locations of nucleosome IV and the α2 operator in the TALS minichromosome are schematically shown on the left of the gel. The horizontal dashed lines indicate the regions corresponding to nucleosome IV in the TALS minichromosomes.

To clarify the alterations of nucleosome positioning by the telomeric repeat insertions in more detail, we performed primer extension mapping of the MNase cleavage sites at base-pair resolution (Figure 3 and Supplementary Figure S1). The effects of the insertions on the nucleosome positioning were monitored by the cleavage sites in the naked DNA (the bands marked with *a, *b and *c) and the cleavage of the inserted repeats in the region of nucleosome IV. A region encompassing ∼140 bp of nucleosome IV was protected against MNase digestion in the TALS chromatin sample (Figure 3, lane 1) as well as the hTEL2 chromatin (Figure 3, lane 3), as compared with the digestion of their naked DNAs (Figure 3, lanes 2 and 4), indicating that 12 bp of human telomeric repeats were incorporated in the positioned nucleosome IV. For the hTEL4 chromatin (Figure 3, lane 5), the cleavage sites indicated by *a-*c were less protected, as compared with those of the hTEL2 chromatin (Figure 3, lane 3), and the region of the hTEL4 insert became accessible to MNase. This implied that the positioning of nucleosome IV is partially destabilized by the insertion of the 24-bp repeats. These subtle changes by the hTEL4 insertion could not be detected clearly by indirect end-labelling; therefore, primer extension mapping was used as a more sensitive assay with higher resolution.

As the repeat length increased to ≧36 bp (hTEL6, hTEL12, hTEL29, yTEL4.5 and yTEL9), the MNase cleavage sites indicated by *a, *b and *c in the region of nucleosome IV were prominently detectable in their chromatin samples to the same extent as in the naked DNA samples. Furthermore, MNase cleavage within the inserts of hTEL6, hTEL12 and hTEL29 was detectable at a similar level between the chromatin (Figure 3, lanes 7, 15 and 23) and the naked DNA samples (lanes 8, 16 and 24). In contrast, in the minichromosomes containing the non-telomeric sequence isomers (SI-A6, SI-A12, SI-B6 and SI-B12), the nucleosome IV regions including the sites with *a, *b and *c and the inserted repeats were protected from MNase digestion (Figure 3, lanes 11, 13, 19 and 21). Thus, taken together with the end-label mapping results, our analyses clearly showed that the insertions of human and yeast telomeric repeats longer than 36 bp disrupt the formation of the positioned nucleosome IV.

### Effect of the telomeric repeats on nucleosome spacing

**Figure 4.**
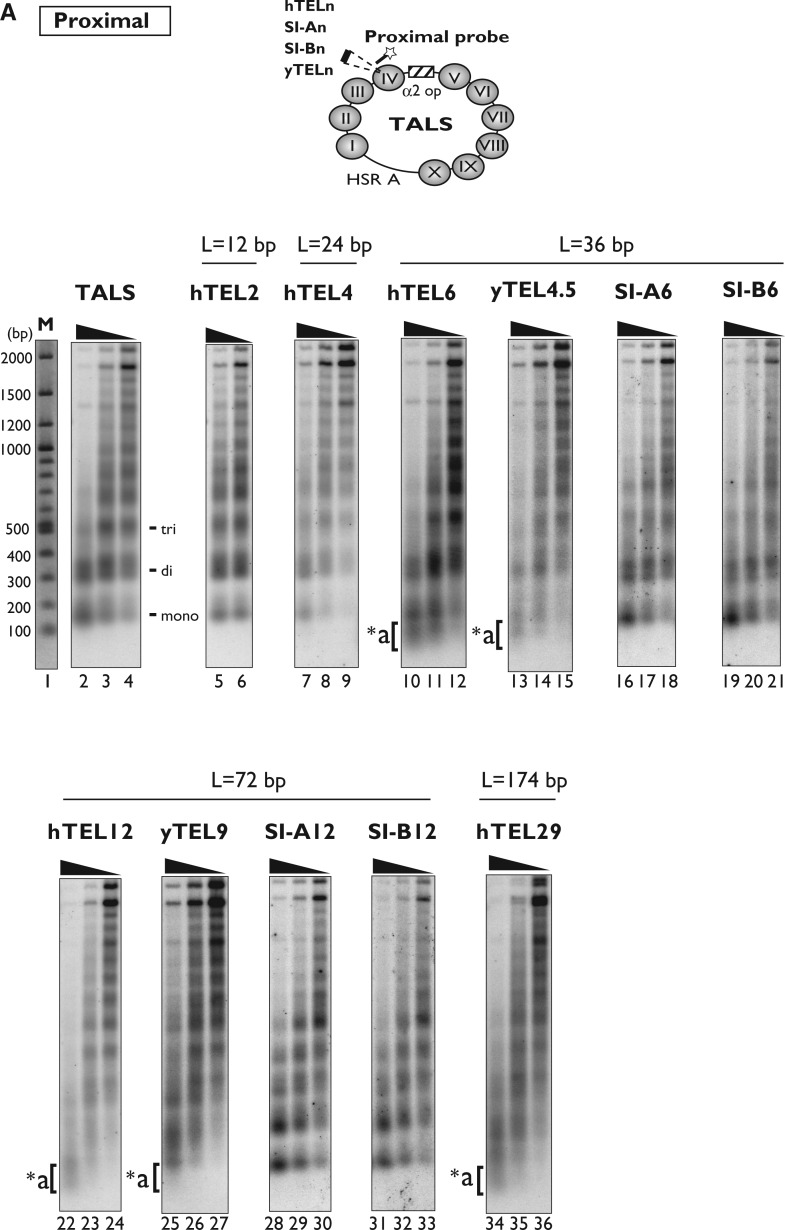

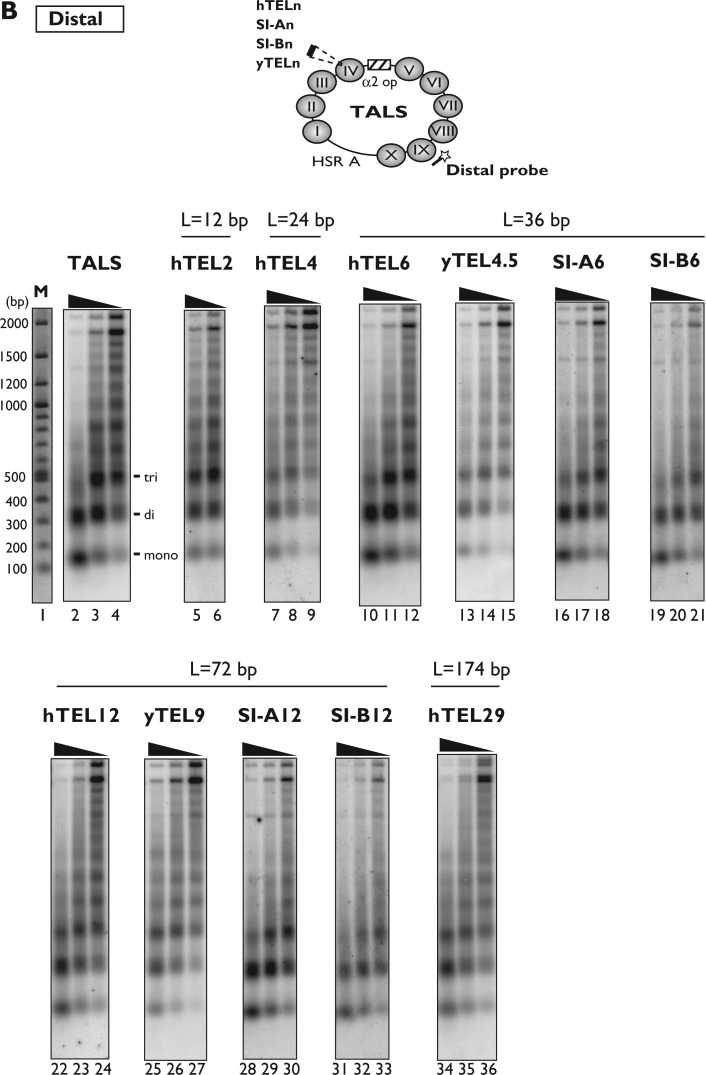
Nucleosome repeat analysis of TALS and its derivative minichromosomes. DNAs from MNase-digested chromatin samples (isolated nuclei) were fractionated by 1.3% agarose gel electrophoresis, and the samples on the gel were transferred to a nylon membrane for Southern blotting. The membrane was probed with ^32^P-labelled proximal (A) or distal (B) probes (see Materials and Methods, and Results). The locations of the proximal and distal probes are schematically drawn on the TALS minichromosome. The lane labelled with ‘M’ indicates DNA markers, which were visualized by ethidium bromide staining on the same agarose gel used for the nucleosome repeat assay.

We further examined the effect of the telomeric repeats on the nucleosome spacing in the minichromosomes, using the nucleosome repeat assay. In this assay, Southern blots of nucleosome ladders produced by MNase digestion were hybridized with probes that are proximal or distal to the nucleosome IV region. Figure 4A shows the results of the nucleosome repeat assay, detected using a proximal probe containing a complementary sequence (1513–1467 nt) near the DNA insertion site in the nucleosome IV region, which was successfully used in a similar previous analysis (26). The nucleosome ladders of the minichromosomes containing the shorter telomeric repeats (hTEL2 and hTEL4, lanes 5–9 in Figure 4A) were quite similar to that of the vector TALS minichromosomes (lanes 2–4), indicating that the hTEL2 (12 bp) and hTEL4 (24 bp) repeats were incorporated into nucleosome IV in the array of nucleosomes with regular spacing.

In contrast, the nucleosome ladders were smeared for the minichromosomes containing the 36-bp telomeric repeats (hTEL6 and yTEL4.5, lanes 10–15 in Figure 4A), as compared with the control insert SI-A6 and SI-B6 (36 bp) minichromosomes (lanes 16–21), and significant amounts of MNase-digested fragments shorter than the mono-nucleosomal DNA band were detected (marked with *a, lanes 10–15). For the minichromosomes containing the 72-bp telomeric repeats (hTEL12 and yTEL9, lanes 22–27), the nucleosome ladders became more smeared, and the shorter fragments (marked with *a) were also detectable. In contrast, the control SI-A12 and SI-B12 (72 bp) minichromosomes showed clear nucleosome ladders (Figure 4A, lanes 28–33), indicating that the insertions of the control sequence isomers did not affect the regular spacing in the nucleosome arrays. It should be noted that for the SI-A12 and SI-B12 minichromosomes, the ladders of mono- and dinucleosomes appeared more discretely, as compared with those of the TALS minichromosome (lanes 2–4), indicating that the nucleosomes are more tightly packed in the SI-A12- and SI-B12-containing minichromosomes. This is consistent with the indirect end-label mapping results (Figure 2, lanes 29–34), in which one additional nucleosome was present in the region between nucleosomes III and IV.

Similar to the hTEL12 minichromosome (Figure 4A, lanes 22–24), a smeared pattern of nucleosome ladders was observed for the hTEL29 minichromosome (lanes 34–36). The smearing of the nucleosome ladders indicated that the nucleosome IV regions near the inserts completely lost their spacing periodicity. The shorter fragments marked with *a were attributed to the extensive digestion of the nucleosome IV region with MNase, implying that the insertion of hTEL6, hTEL12, hTEL29, yTEL4.5 and yTEL9 prevented the histone octamers from binding to the nucleosome IV region.

Using the same method, the nucleosome spacing in the TALS and its derivative minichromosomes distal to the nucleosome IV region was examined, by hybridizing the blots with a distal probe containing a sequence within the nucleosome IX region (381–497 nt). As shown in Figure 4B, the nucleosome ladders from all of the minichromosomes appeared identical to the canonical nucleosome ladders, demonstrating that the perturbation of the nucleosome spacing near the nucleosome IV region, by the insertion of telomeric repeats, does not propagate into the distal nucleosome IX region.

## DISCUSSION

Using the defined yeast minichromosome system, we examined the effects of telomeric repeat sequences on the formation of nucleosomes *in vivo*, by three different analyses (indirect end-label mapping, high-resolution primer extension mapping and nucleosome repeat assay). Previous studies showed that telomeric repeat regions assume a non-nucleosomal chromatin structure in *S. cerevisiae* (3,4,7), whereas they are organized in tightly packaged nucleosomes in human (10,12). However, we found here that the human and yeast telomeric repeats have similar properties, as they both disfavour nucleosome formation in the yeast minichromosomes *in vivo*.

In principle, there are two possible mechanisms for the alterations of nucleosome assembly and positioning by the longer telomeric repeats. One is the occupation of the repeat by yeast DNA binding proteins, such as the TTAGGG-binding protein factor 1 (Tbf1) and the yeast telomeric binding protein Rap1. For the insertion of human telomeric repeats in the minichromosomes, the two and four binding sites in hTEL2 and hTEL4 might be insufficient for Tbf1 binding to compete with nucleosome formation, but ≥ 6 binding sites (hTEL6 or more) are sufficient. However, we believe this is unlikely to occur because Tbf1 reportedly functions by binding to a 5'-aRCCCTaa-3' site, which corresponds to one human telomeric repeat, in the upstream regions of many small nucleolar RNA genes (52). Furthermore, although one or two human telomeric repeats are present, repeats of three or more have not been detected in the *S. cerevisiae* genome. Rap1 would not be involved in the mechanism for the nucleosome-disfavouring properties of the human telomeric repeats, as it does not bind to them (53,54). For the insertion of yeast telomeric repeats, Rap1 could compete with the histone octamers to bind to yTEL4.5 and yTEL9 (53,54).

The other mechanism would be the inherent structural features of the telomeric repeat sequences. To clarify the sequence requirements in the molecular mechanism, we analysed the sequence isomers of TTAGGG, (TGTAGG)_n_ and (TGTGAG)_n_, in which the three consecutive G residues, as a defining characteristic of the telomeric repeat, are dispersed, while the same base composition is maintained. The inserts of the sequence isomers with lengths of 36 and 72 bp were both incorporated into the positioned nucleosome IV, whereas the hTEL6 (36 bp) and hTEL12 (72 bp) inserts were not. Thus, the G-cluster in the telomeric repeats is essential for the nucleosome-disfavouring properties.

**Figure 5.**
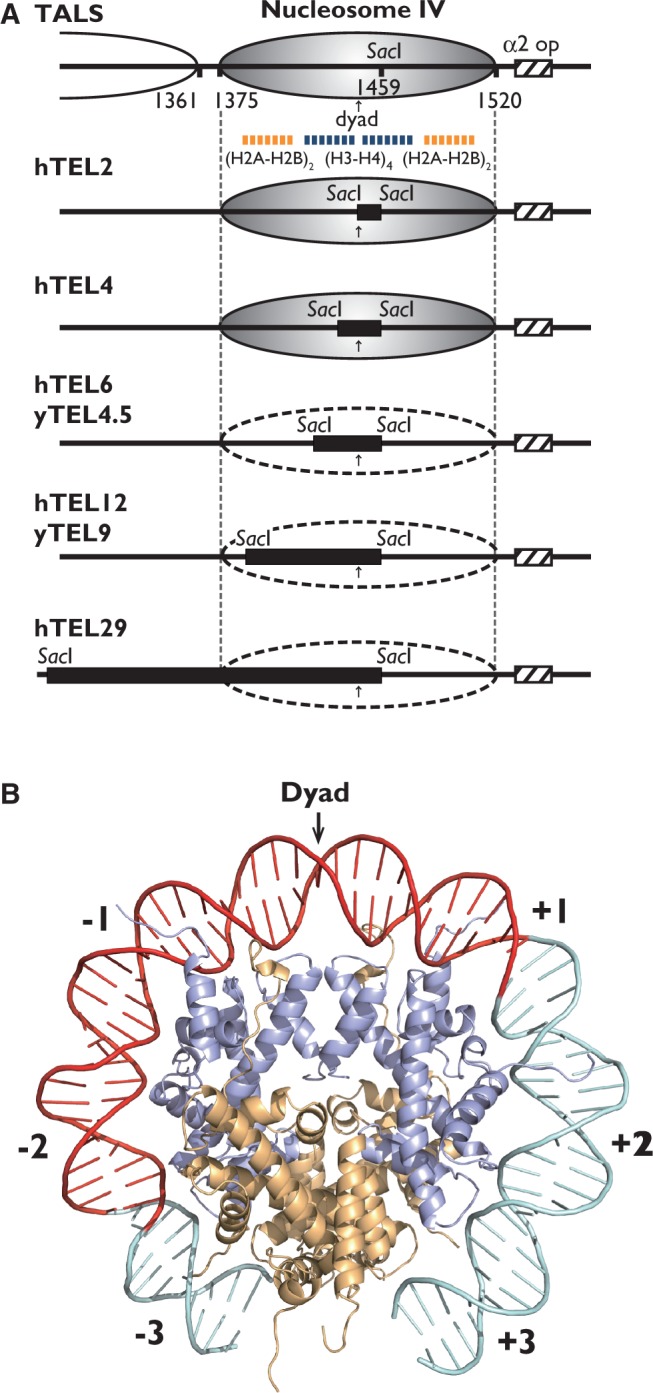
(**A**) Summary of the effect of telomeric repeats on nucleosome formation in the TALS minichromosomes. Solid and dotted ellipses indicate the presence and absence of the positioned nucleosome IV, respectively. Insertions of the telomeric repeats (hTEL2, hTEL4, hTEL6, hTEL12 and hTEL29 for humans, and yTEL4.5 and yTEL9 for yeasts) and the α2 operator are indicated by black and hatched boxes, respectively. The DNA regions interacting with the histone H3–H4 tetramer and the H2A–H2B dimers are shown by blue and orange dashed lines, respectively. (**B**) A projection of 36 bp of the telomeric repeats (hTEL6 or yTEL4.5) on the crystal structure of the yeast nucleosome core particle (PDB: 1ID3), which was determined by White *et al.* (29). The DNA path from SHL −3 to +3 is shown on the structure, and DNA strands (red) show 36 bp of the hTEL6 or yTEL4.5 region. Histone chains are shown in orange for H2A and H2B, and blue for H3 and H4.

As shown in Figure 5A, the hTEL2 and hTEL4 inserts were incorporated in nucleosome IV and were located near the pseudo-dyad axis. On the other hand, the positioning of nucleosome IV was disrupted in the minichromosomes containing longer hTEL12, yTEL9 or hTEL29 sequences. We then focused on hTEL6 and yTEL4.5, which were relatively short but were not incorporated in nucleosome IV in the TALS minichromosome. The locations of their sequences were projected on the DNA path of the yeast nucleosome crystal structure, which was determined by White *et al.* (29) (Figure 5B). The DNA path of these repeats (36 bp) would span from superhelix axis location (SHL)  +1 to −2.5, containing four minor groove contact sites (SHL +0.5, −0.5, −1.5 and −2.5). These sites involve interactions with the H3–H4 tetramer and part of the H2A–H2B dimer. In contrast, the path of the hTEL4 repeat (24 bp) would span from SHL +1 to −1 with two contact sites (SHL +0.5 and −0.5), and involve interactions with the H3–H4 tetramer. Thus, the bendability of the telomeric repeats may become more restricted as the repeat length increases. This idea is consistent with the previous reports that the DNA structures containing consecutive G residues are quite unusual. The d(G-G-G-G) segments in the d(G-G-G-G-C-C-C-C) crystal structure form the A-type double helix (55). In the aqueous DNA duplex of this octamer, the guanine–guanine stacking is A-like (56). Thus, owing to the A-like structural properties of the G-clusters (55–57), telomeric repeats longer than 36 bp may be too rigid to wrap around the histone octamer, particularly on the DNA path of SHL −1 to −2.5, which is the region predicted to be spanned by hTEL6 and yTEL4.5, but not hTEL4. In terms of the molecular mechanism, the periodic appearance of the G-clusters in the repeats disfavours nucleosome formation.

Alternatively, telomeric repeat sequences are known to form G-quadruplexes (G4) (4), and hence G4 formation may prevent nucleosome assembly on the telomeric repeats. A model has been proposed for the stalling of replication forks at telomeric repeats, due to G4 folding (58). However, recent studies revealed that DNA polymerase δ stalls on telomeric lagging strands independently from G4 formation (59). Thus, it is uncertain whether G4 folding occurs during replication at telomeric repeats inserted in the TALS minichromosome, and if so, whether the G4 structures are involved in the nucleosome-disfavouring properties of telomeric repeats.

Previous *in vitro* studies revealed that the free energies for nucleosome formation of telomeric repeat sequences from several organisms, including human and yeast, were comparable, and they are the lowest among the various sequences tested (4,30–32). Most telomeric repeats are predicted to be linear (4,11), and thereby would require more energy for bending to form nucleosomes. This idea is consistent with our results that longer repeats suppress nucleosome formation, as discussed above. In addition, *in vitro* DNase I and lambda exonuclease footprinting studies showed that nucleosomes occupy multiple positions on the telomeric repeats (31). An AFM imaging analysis revealed that the spacing between nucleosomes on 800–1500 bp of human telomeric repeats varied randomly, whereas the nucleosomes on the 5S ribosomal DNA and the 601 sequences were uniformly spaced (34,60). These studies clearly demonstrated that the nucleosomes formed on the human telomeric repeats are labile, and highly intrinsically mobile. Our finding that the telomeric repeats act as nucleosome-disfavouring sequences *in vivo* is consistent with the features of nucleosomes reconstituted from the human telomeric repeats *in vitro*.

Nonetheless, tightly packaged nucleosomes are formed in the tandem repeats of human telomeres. In previous studies, Southern blots of MNase-digested chromatin were probed with telomeric repeat sequences (10,12), which is a typical method for analysing the nucleosome organization of bulk chromatin. Hence, if the fraction of the nucleosome-free region were smaller, then it would be harder to detect. In contrast, our approach included a detailed analysis for properly positioned nucleosomes without human telomeric factors in yeast cells, and thereby the effects of the DNA properties of the telomeric repeats could be solely examined *in vivo*.

In human telomeres, TRF1 and TRF2 recognize GGGTTAGGG in the two repeats (16–18) and they bind to 20–30% and ∼15% of all repeat regions, respectively (61). Furthermore, TRF1 and TRF2 form a complex with telomeric nucleosomes, and alter their structures (19–24). Because telomeric repeat regions are much longer (several 1000 bp) in higher eukaryotes, as compared with lower eukaryotes, it would be unfavourable to have a long nucleosome-free region in telomeres, in terms of chromosomal stability. Thus, higher eukaryotes may have evolved to develop specific and functional chromatin structures including nucleosomes. Such development may also have occurred in lower eukaryotes, as 3–10% of the telomere region in the lower eukaryote *Tetrahymena* is organized in nucleosomes (62). The inherent feature of telomeric repeats to disfavour nucleosome formation, in concert with telomeric binding proteins (such as TRF1 and TRF2), telomere-specific histone variants, histone modifications and histone chaperones (63–67), may play a crucial role in the formation and dynamics of the telomeric chromatin structure.

## SUPPLEMENTARY DATA

Supplementary Data are available at NAR Online.

## FUNDING

The Ministry of Education, Culture, Sports, Science and Technology (MEXT), Japan (in part to M.S., Y.N. and H.K.); Japanese Society for the Promotion of Science (JSPS) (to M.S. and H.K.); Supported by the Waseda Research Institute for Science and Engineering (to H.K.). Funding for open access charge: Waseda University.

*Conflict of interest statement*. None declared.

## Supplementary Material

Supplementary Data
