# Supplementary material for: Telomeric repeats act as nucleosome-disfavouring sequences in vivo
Source: Nucleic Acids Res. 2013 Oct 29;42(3):1541–52. doi: 10.1093/nar/gkt1006 (PMC3919577; doi:10.1093/nar/gkt1006)
Supplement: Supplementary Data [file supp_42_3_1541__index.html]

Telomeric repeats act as nucleosome-disfavouring sequences in vivo — Telomeric repeats act as nucleosome-disfavouring sequences in vivo — Supplementary Data 

# Telomeric repeats act as nucleosome-disfavouring sequences *in vivo*

## Supplementary Data

files

**Files in this Data Supplement:**

- Supplementary Data - pdf file
