# Supplementary material for: Telomeric repeats act as nucleosome-disfavouring sequences in vivo
Source: Nucleic Acids Res. 2013 Oct 29;42(3):1541–52. doi: 10.1093/nar/gkt1006 (PMC3919577; doi:10.1093/nar/gkt1006)
Supplement: Supplementary Data [file supp_gkt1006_nar-02085-m-2013-File008.pdf]

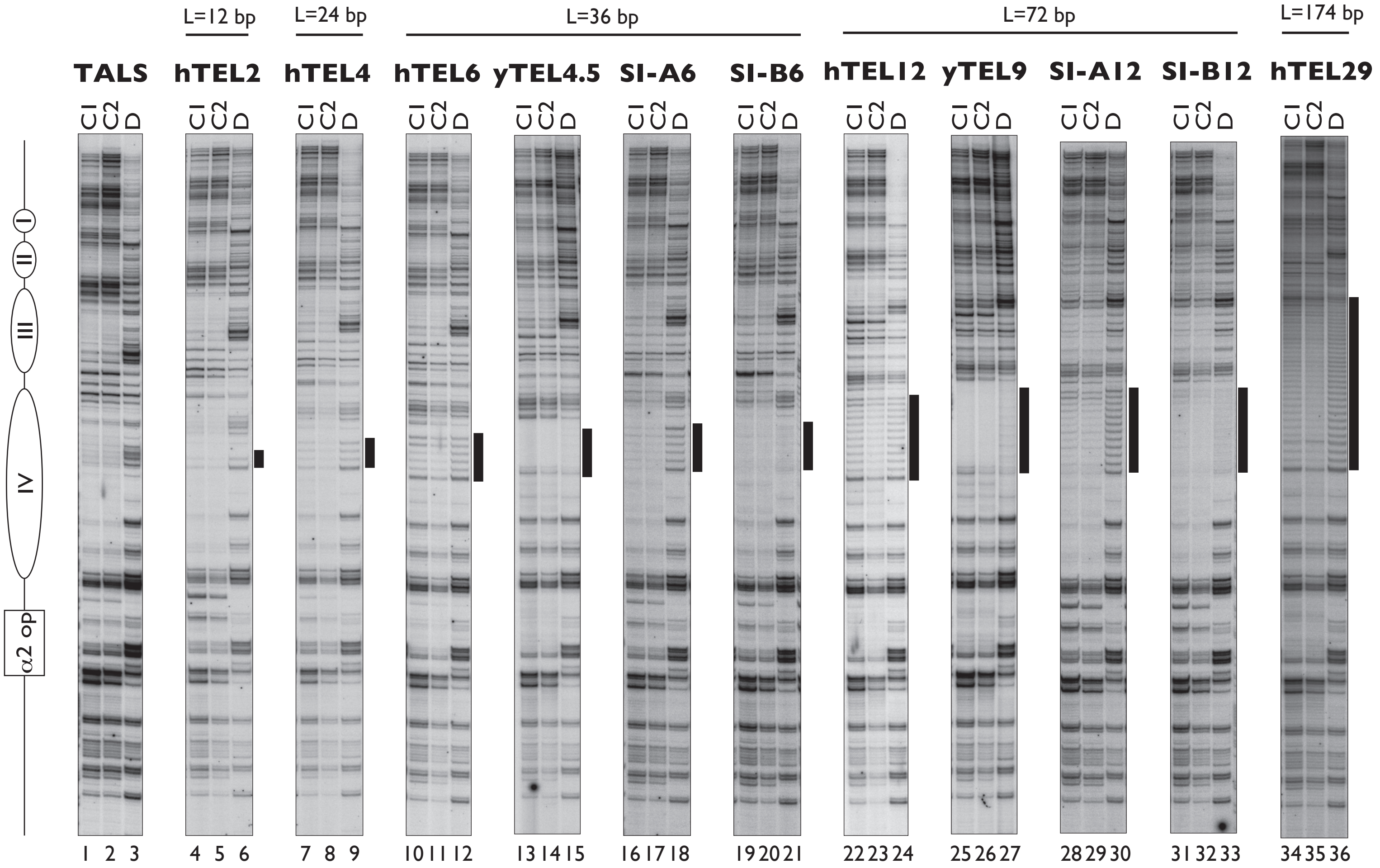

Supplementary Figure S1. Whole data for the high resolution primer extension mapping of MNase cleavage sites in TALS and its derivatives containing human and yeast telomeric repeats or sequence isomers. Lanes labelled with “C1” and “C2” indicate MNase digestion of isolated nuclei at two nuclease concentrations, and lanes labelled with “D” indicate digestion of the naked DNA as a control. The locations of the DNA inserts are indicated by black boxes. The locations of nucleosomes I to IV and the α2 operator in the TALS minichromosome are schematically shown on the left of the gel.

Ichikawa *et al.*, Figure S1
